# Disparities in breast cancer diagnosis for immigrant women in Ontario and BC: results from the CanIMPACT study

**DOI:** 10.1186/s12885-018-5201-0

**Published:** 2019-01-09

**Authors:** A. K. Lofters, M. L. McBride, D. Li, M. Whitehead, R. Moineddin, L. Jiang, E. Grunfeld, P. A. Groome, for the CanIMPACT Team, Natalie Biswanger, Kathleen Decker, Cynthia Kendell, Monika Krzyzanowska, Sharon Nicole Mittmann Matthias, Geoff Porter, Dawn Powell, Donna Turner, Bonnie Vick, Marcy Winget, Yan Yuan

**Affiliations:** 1 Department of Family & Community Medicine, St. Michael’s Hospital, 30 Bond St, Toronto, M5B 1W8 Canada; 2 Centre for Urban Health Solutions, Li Ka Shing Knowledge Institute, Toronto, Canada; 3 Department of Family & Community Medicine, University of Toronto, Toronto, Canada; 4 Dalla Lana School of Public Health, University of Toronto, Toronto, Canada; 5 ICES, Toronto, Canada; 6 BC Cancer, Vancouver, Canada; 7 School of Population and Public Health, University of British Columbia, Vancouver, Canada; 8 ICES, Queen’s University, Kingston, Canada; 9 Critical Care Services Ontario, Toronto, Ontario Canada; 10 Ontario Institute for Cancer Research, Toronto, ON Canada; 11 Department of Public Health Sciences, Queen’s University, Kingston, Canada; 12 Cancer Research Institute, Queen’s University, Kingston, Canada

**Keywords:** Immigrant health, Breast cancer, Stage of diagnosis, Health equity, Diagnosis interval, Cancer screening

## Abstract

**Background:**

In Canada, clinical practice guidelines recommend breast cancer screening, but there are gaps in adherence to recommendations for screening, particularly among certain hard-to-reach populations, that may differ by province. We compared stage of diagnosis, proportion of screen-detected breast cancers, and length of diagnostic interval for immigrant women versus long-term residents of BC and Ontario.

**Methods:**

We conducted a retrospective cohort study using linked administrative databases in BC and Ontario. We identified all women residing in either province who were diagnosed with incident invasive breast cancer between 2007 and 2011, and determined who was foreign-born using the Immigration Refugee and Citizenship Canada database. We used descriptive statistics and bivariate analyses to describe the sample and study outcomes. We conducted multivariate analyses (modified Poisson regression and quantile regression) to control for potential confounders.

**Results:**

There were 14,198 BC women and 46,952 Ontario women included in the study population, of which 11.8 and 11.7% were foreign-born respectively. In both provinces, immigrants and long-term residents had similar primary care access. In both provinces, immigrant women were significantly less likely to have a screen-detected breast cancer (adjusted relative risk 0.88 [0.79–0.96] in BC, 0.88 [0.84–0.93] in Ontario) and had a significantly longer median diagnostic interval (2 [0.2–3.8] days in BC, 5.5 [4.4–6.6] days in Ontario) than long-term residents. Women from East Asia and the Pacific were less likely to have a screen-detected cancer and had a longer diagnostic interval, but were diagnosed at an earlier stage than long-term residents. In Ontario, women from Latin America and the Caribbean and from South Asia were less likely to have a screen-detected cancer, had a longer median diagnostic interval, and were diagnosed at a later stage than long-term residents. These findings were not explained by access to primary care.

**Conclusions:**

There are inequalities in breast cancer diagnosis for Canadian immigrant women. We have identified particular immigrant groups (women from Latin America and the Caribbean and from South Asia) that appear to be subject to disparities in the diagnostic process that need to be addressed in order to effectively reduce gaps in care.

## Background

Women in Canada are more likely to develop breast cancer than any other cancer, with 1 in 8 Canadian women expected to develop the disease in their lifetime, and 1 in 31 expected to die from it [1]. Diagnosing cancer at an early stage is one of the most important determinants of a positive outcome, and delays in diagnosis have been associated with disease progression (and thus higher stage at the time of diagnosis) and inefficient healthcare delivery [2–4]. In Canada, clinical practice guidelines recommend mammography for breast cancer screening, but there are province-specific gaps in adherence to recommendations for screening, particularly among certain hard-to-reach populations, and many women are still diagnosed due to symptomatic presentation [5–7].

There are known breast cancer screening inequalities in Canada based on sociodemographic characteristics. In particular, some immigrant women have been found to have lower rates of mammography use than Canadian-born women [6, 9–13]. As well, ethnicity has been associated with stage at breast cancer diagnosis in the province of Ontario, with women of South Asian ethnicity being more likely to be diagnosed at a later stage, and women of Chinese ethnicity being more likely to be diagnosed at an early stage, than the remaining general population [14]. Little is known about the role of sociodemographic characteristics in the length of the diagnostic interval in Canada, although large variation in the length of the diagnostic interval has been observed based on geography [15]. Much remains to be learned about potential disadvantage for immigrants as it relates to a timely breast cancer diagnosis.

As immigrant women from South Asia have been found to have particularly low breast cancer screening rates [6], we hypothesized that women from this region would also have a later stage of diagnosis and a lower proportion of screen-detected breast cancers. We further hypothesized that immigrant class i.e. one’s classification upon admission to the country and time in the country would be associated with the diagnostic process based on their association with screening uptake in previous studies [6, 16]. As ethnicity has been linked to the length of the breast cancer diagnostic interval in the literature [17], we also wanted to explore the relationship of the aforementioned immigrant-related variables with the length of the diagnostic interval. Comparing population-level results on cancer care across provincial/territorial healthcare delivery systems would identify important similarities and differences between jurisdictions, and contextual differences that might affect the gaps in care [8]. The identification of immigrant factors that are related to lower screening rates and longer diagnostic intervals could help guide outreach efforts to improve access.

Together, the provinces of British Columbia (BC) and Ontario receive more than half of all immigrants to Canada [18]. Both provinces have organized breast screening programs that recommend biennial screening with mammography for average-risk women aged 50–74 years [5, 7]. In this study, we aimed to compare: i) stage of diagnosis, ii) the proportion of screen-detected breast cancers, and iii) the length of the diagnostic interval for immigrant women versus long-term residents of BC and Ontario. We explored differences within immigrants by characterizing them in three ways: by region of origin, immigrant class, and time in Canada. .

## Methods

### Study design

This study was conducted as part of the Canadian Team to Improve Community-Based Cancer Care along the Continuum (CanIMPACT), a multi-province multi-method program of research aimed at improving integration and coordination of care along the cancer care continuum [19]. We conducted a retrospective cohort study using linked administrative databases in two of the CanIMPACT participating provinces: Ontario and BC.

### Data sources

Data sources for both provinces included the provincial cancer registries, health insurance plan patient registry data, physician billing claims data, and the federal government’s Immigration Refugee and Citizenship Canada (IRCC) database. We used the provincial cancer registries (BC Cancer Registry and Ontario Cancer Registry) to identify the study cohort and to determine date of cancer diagnosis, detection method and cancer stage [20]. Patient registry data (BC Medical Services Plan Client Registry and Ontario Registered Persons Database) were used to obtain age, sex and postal code. Billing claims data (BC Medical Services Plan Claims Database and Ontario Health Insurance Plan data) captured outpatient physician services covered under the two provinces’ universal health insurance plans [21, 22]. The IRCC database was used to identify Canadian immigrants who arrived from 1985 onward, and includes detailed demographic information such as country of birth, immigrant class, and date of achieving permanent residency status for those who could be either deterministically or probabilistically linked to existing databases [23]. Immigrant class refers to the IRCC classification of immigrants upon admission to the country, specifically: economic (those selected on the basis of their ability to become economically established in Canada), family (those sponsored by a family member), and refugee (those fleeing their countries because of fear of persecution). These provincial population-level administrative databases were linked using unique encoded identifiers and analysed at the ICES in Ontario and Population Data in BC. The study was approved by the Health Sciences and Affiliated Hospitals Research Ethics Board at Queen’s University in Ontario, and the BC Cancer Agency and University of British Columbia Research Ethics Board in British Columbia. Data access approvals were obtained from all data stewards in each province. Patient consent was deemed as not required because the research involved no more than minimal risk, the research did not involve an intervention, lack of consent was unlikely to adversely affect patient welfare, and it was impracticable to obtain consent. Due to data access and use constraints which do not readily permit data to leave the respective provinces [24], the analysis was conducted separately in each province.

### Study population

We identified all women residing in Ontario or BC who were diagnosed with incident invasive breast cancer between January 1, 2007 and December 31, 2011. Women were excluded if they did not have a valid health card number or were living outside of the province at the time of diagnosis. Both provinces have universal health insurance whereby all permanent residents are eligible for a health card i.e. for medically necessary health care [25]. Women were also excluded if they had a history of in situ breast cancer, any non-melanoma cancer, or non-solid breast tumour.

The IRCC database was used to identify women who were immigrants to Canada since 1985, and to determine their country of birth, immigrant class, and permanent landing date. Countries of birth were then classified into world regions based on a modified version of the World Bank classification system [6, 26–30]. Women who were not in the IRCC database were classified as “long-term residents”, as some of these women would be Canadian-born and some would be earlier immigrants who had arrived prior to 1985. Women’s immigrant class was categorized as economic, family, refugee or other, depending on their reason for immigration to Canada. Landing date was used to classify women as having been in Canada for less than 10 years or for 10 years or more.

### Study outcomes

We considered three outcomes: cancer stage at diagnosis (I-IV) [31, 32], whether the breast cancer was screen-detected or not, and the length of the diagnostic interval. The diagnostic interval was defined as the time from the date of the first cancer-related physician encounter, diagnostic investigation or screening date to the date of breast cancer diagnosis.

### Study variables

We retrieved data on specific possible modifiers of risk, prioritized in the CanIMPACT program, namely co-morbidities, income and age at diagnosis. These factors have been associated with breast cancer screening uptake in Canada [6, 16, 33]. Co-morbidities were defined using Aggregated Diagnosis Groups (ADGs) from the Johns Hopkins Adjusted Clinical Groups (ACG) Case Mix System, which identifies morbidities from diagnosis codes in outpatient billing and inpatient (hospital) records [34]. The ADGs represent groups of conditions with similar healthcare experience relating to attributes such as severity and duration of disease; multimorbidity was defined as the number of ADGs per individual at diagnosis. Neighbourhood income quintile was defined based on a woman’s residential postal code at the time of diagnosis, whereby Canadian 2006 Census household income data was used to create community-specific income quintiles [35]. Age at diagnosis was obtained from the aforementioned provincial registries.

As breast cancer screening is often recommended and/or ordered by a primary care physician, and as primary care physicians often play a key role in breast cancer diagnosis, we considered primary care access to be on the causal pathway. We did not include primary care access as a confounder, but we did collect primary care data. Continuity of primary care was defined based on the baseline Usual Provider of Care (UPC) index i.e. the proportion of all primary care visits during the 6 to 30 months before diagnosis that were made to the primary care provider most frequently visited (among those women with at least 3 primary care visits in that time period). We also quantified the number of visits to a primary care provider during the 6 months before diagnosis.

### Analyses

We used descriptive statistics and bivariate analyses to describe the sample and the study outcomes. We also conducted multivariate analyses to control for potential confounders. Modified Poisson regression was used to estimate relative risk for stage of diagnosis, which was dichotomized to stage I-II versus stage III-IV, and for cancers that were screen-detected versus not. We dichotomized stage in this manner as 5-year relative survival drops from 93 to 72% for stage II versus stage III [36]. Quantile regression was used to explore differences in the median of the diagnostic interval. Variables included in multivariate analyses were age at diagnosis, multimorbidity, neighbourhood income quintile, and immigrant status. Quantile regression analyses also included stage at diagnosis, as the diagnostic interval tends to be shorter for later stage disease.

To explore which immigrant characteristics were associated with our outcomes, all models were repeated, with immigrant women stratified by each of: region of origin, immigrant class and time in Canada. These variables were not put into one model due to concerns about multicollinearity and interpretation of adjusted results. To justify this stratified approach, post hoc, multivariable regression models were conducted in both provinces to assess for the presence of interaction between region of origin, immigrant class, and time in Canada.

## Results

There were 14,198 BC women and 46,952 Ontario women included in the study population. Immigrants made up 11.8 and 11.7% of the provincial cohorts respectively (Table 1). East Asia and the Pacific was the most common source region for immigrants in both provinces, representing 6.7 and 3.5% of the respective cohorts. The economic class was the most common immigration class in both provinces, but the proportion of refugees in Ontario was approximately twice that of BC. The majority of immigrants had been in Canada for at least 10 years.

**Table 1:** Immigration-related characteristics of the study population in BC and Ontario

		BC	Ontario
	N (%)	14,198 (100)	46,952 (100)
	Long-term residents	12,520 (88.2)	41,457 (88.3)
	Immigrants	1678 (11.8)	5495 (11.7)
Region	East Asia & Pacific	948 (6.7)	1636 (3.5)
	Eastern Europe & Central Asia	121 (0.9)	933 (2.0)
	Latin American & Caribbean	64 (0.5)	752 (1.6)
	Middle East & North Africa	93 (0.7)	513 (1.1)
	South Asia	191 (1.4)	968 (2.1)
	Sub-Saharan Africa	56 (0.4)	261 (0.6)
	USA/New Zealand/Australia	68 (0.5)	121 (0.3)
	Western Europe	137 (1.0)	311 (0.7)
Immigrant Class	Economic	961 (6.8)	2496 (5.3)
	Family	609 (4.3)	2190 (4.7)
	Refugee	94 (0.7)	699 (1.5)
	Other	14 (0.1)	110 (0.2)
Years since immigration	< 10 years	613 (4.3)	1981 (4.2)
	> = 10 years	1065 (7.5)	3514 (7.5)

Characteristics of women with breast cancer varied between the two provinces and between regions of origin (Table 2, Table 3). In both provinces, immigrant women had a similar mean number of primary care visits in the six months prior to diagnosis versus long-term residents (3.3 + 2.9 for long-term residents vs. 2.9 + 2.4 for immigrants in BC, mean 3.4 + 3.3 for long-term residents vs. 4.0 + 3.2 for immigrants in Ontario), but were less likely to have a high UPC index. In both provinces, immigrant women were younger at diagnosis. In BC, women from Eastern Europe and Central Asia (29.8%) and from South Asia (27.8%) were most represented in the lowest income quintile, as compared to Ontario where women from Latin America and the Caribbean (30.9%) and from Sub-Saharan Africa (34.9%) were most represented in the lowest income quintile. Ontario immigrants generally had more co-morbidities than those in BC, and screen-detected breast cancers were less prevalent in Ontario than in BC. In both provinces, immigrant women had fewer screen-detected breast cancers and a lower prevalence of a Stage I diagnosis than long-term residents. South Asian women in both provinces had a particularly low prevalence of Stage I diagnosis (26.7% for BC, 25.8% for Ontario). Ontario women from Latin America and the Caribbean had the lowest probability of being screen-detected overall (17.4%).

**Table 2:** Characteristics of the study population in BC, stratified by immigrant status and region of origin

Variable	Value	Long-term residents	Immigrants	East Asia & Pacific	Eastern Europe & Central Asia	Latin America & Caribbean	Middle East & North Africa	South Asia	Sub-Saharan Africa	USA/New Zealand/Australia	Western Europe
N (%)		12,520 (88.2)	1678 (11.8)	948 (6.7)	121 (0.8)	64 (0.5)	93 (0.7)	191 (1.4)	56 (0.4)	68 (0.5)	137 (1.0)
Primary care access variables											
Baseline UPC index score	0 visits	933 (7.5)	207 (12.3)	117 (12.3)	17 (14.1)	<=5	16–20	12 (6.3)	6–10	6–10	19 (13.9)
	1–2 visits	1694 (13.5)	0 (0)	153 (16.1)	17 (14.1)	<=5	<=5	19 (10.0)	<=5	<=5	24 (17.5)
	UPC < =.75 (low)	4641(37.1)	591 (35.2)	322 (34.0)	42 (34.7)	25 (39.06)	36 (38.7)	69 (36.1)	19 (33.9)	24 (35.3)	54 (39.4)
	UPC > 0.75 (high)	5252 (42.0)	638 (38.0)	356 (37.6)	45 (37.2)	28 (43.75)	29 (31.2)	91 (47.6)	21 (37.5)	28 (41.2)	40 (29.2)
Primary care visits in 6 mths prior to diagnosis	Mean ± SD	3.3 ± 2.9	2.9 ± 2.4	2.8 ± 2.4	2.9 ± 2.4	2.6 ± 2.0	2.7 ± 2.4	2.8 ± 2.9	2.5 ± 1.9	3.0 ± 2.7	2.7 ± 2.4
	Median (IQR)	3 (1–4)	2 (1–4)	2 (1–4)	2 (1–4)	3 (1–4)	2 (0–5)	3 (2–5)	2 (1–4)	3 (1–4)	2 (1–4)
Control variables											
Age at diagnosis	Mean ± SD	63.1 ± 13.6	53.4 ± 12.3	52.6 ± 11.8	54.6 ± 12.1	51. 8 ± 12.0	52.6 ± 10.9	54.6 ± 13.1	52.7 ± 13.0	52.1 ± 11.2	53.7 ± 13.3
Co-morbidities (ADGs)	0 -- 5	6916 (55.2)	1092 (65.1)	649 (68.5)	74 (61.2)	39 (60.9)	58 (62.4)	101 (52.9)	36 (64.3)	38 (55.9)	97 (70.8)
	6 -- 9	4154 (33.2)	468 (27.9)	253 (26.7)	37 (30.6)	21–25	23 (24.7)	65 (34.0)	16–20	21–25	26 (19.0)
	10 +	1450 (11.6)	118 (7.0)	46 (4.9)	10 (8.3)	<=5	12 (12.9)	25 (13.1)	<=5	<=5	14 (10.2)
Income quintile	1 (lowest)	2247 (18.0)	399 (23.8)	245 (25.8)	36 (29.8)	16 (25)	14 (15.1)	53 (27.8)	6–10	14 (20.6)	11–15
	2	2413 (19.3)	389 (23.2)	218 (23.0)	23 (19.0)	11 (17.2)	18 (19.4)	74 (38.7)	6–10	6–10	30 (21.9)
	3	2400 (19.2)	336 (20.0)	189 (19.9)	23 (19.0)	12 (18.8)	18 (19.4)	34 (17.8)	17 (30.4)	14 (20.6)	29 (21.2)
	4	2518 (20.1)	247 (14.7)	139 (14.7)	16 (13.2)	6–10	18 (19.4)	20 (10.5)	6–10	13 (19.1)	23 (16.8)
	5	2707 (21.6)	257 (15.3)	130 (13.7)	17 (14.1)	11–15	18 (19.4)	6–10	16 (28.6)	16 (23.5)	41 (29.9)
	Missing	235 (1.9)	50 (3.0)	27 (2.9)	6 (5.0)	<=5	7 (7.53)	<=5	0 (0)	<=5	<=5
Outcome variabels											
Stage at diagnosis	I	5297 (42.3)	669 (39.9)	396 (41.8)	45 (37.2)	29 (45.3)	36 (38.7)	51 (26.7)	26 (46.4)	26 (38.2)	60 (43.8)
	II	3974 (31.7)	576 (34.3)	329 (34.7)	43 (35.5)	19 (29.7)	25 (26.9)	85 (44.5)	15 (26.8)	23 (33.8)	37 (27.0)
	III	1549 (12.4)	254 (15.1)	123 (13.0)	23 (19.0)	12 (18.8)	19 (20.4)	38 (19.9)	8 (14.3)	9 (13.2)	22 (16.1)
	IV	542 (4.3)	55 (3.3)	21 (2.2)	<=5	<=5	<=5	7 (3.66)	6–10	<=5	7 (5.1)
	Unknown	1158 (9.3)	124 (7.4)	79 (8.3)	<=5	<=5	6–10	10 (5.24)	<=5	6–10	11 (8.03)
Screen-detected	n (%) screen-detected	4470 (35.7)	477 (28.4)	259 (27.3)	33 (27.3)	27 (42.2)	27 (42.2)	61 (31.9)	16 (28.6)	16 (23.5)	35 (25.6)
Diagnostic Interval (Screened patients)	Median (IQR)	32 (17–55)	30 (18–50)	31 (18–50)	41 (30–55)	20 (14–45)	26 (17–44)	31 (16–47)	38 (19–66)	29 (20–51)	28 (19–57)
	90th percentile	81	84	85	87	70	76	73	98	80	80
Diagnostic Interval (Symptom-atic patients)	Median (IQR)	30 (14–63)	34 (15–72)	35 (16–75)	23 (11–57)	38 (17–93)	34 (15–73)	41 (21–74)	23 (11–63)	29 (13–65)	22 (11–69)
	90th percentile	120	141	143	126	173	148	120	187	144	117

*P*-value <.001 for all variables for comparison of immigrants versus long-term residents

**Table 3:** Characteristics of the study population in Ontario, stratified by immigrant status and region of origin

Variable	Value	Long-term residents	Immigrants	East Asia & Pacific	Eastern Europe & Central Asia	Latin American & Caribbean	Middle East & North Africa	South Asia	Sub-Saharan Africa	USA/New Zealand/Australia	Western Europe
N (%)		41,456 (88.3)	5495 (11.7)	1636 (3.5)	933 (2.0)	752 (1.6)	513 (1.1)	968 (2.1)	261 (0.6)	121 (0.3)	311 (0.7)
Primary care access variables											
Baseline UPC index score	0 visits	3022 (7.3)	565 (10.3)	136 (8.3)	103 (11.0)	62 (8.2)	91 (17.7)	91 (9.4)	25 (9.6)	19 (15.7)	38 (12.2)
	1–2 visits	4496 (10.8)	509 (9.3)	156 (9.5)	115 (12.3)	65 (8.6)	33 (6.4)	62 (6.4)	17 (6.5)	18 (14.9)	43 (13.8)
	UPC < =.75(low)	10,703 (25.8)	1796 (32.7)	507 (31.0)	289 (31.0)	270 (35.9)	160 (31.2)	333 (34.4)	109 (41.8)	38 (31.4)	90 (28.9)
	UPC > 0.75(high)	23,235 (56.0)	2625 (47.8)	837 (51.2)	426 (45.7)	355 (47.2)	229 (44.6)	482 (49.8)	110 (42.1)	46 (38.0)	140 (45.0)
Primary care visits in 6 mths prior to diagnosis	Mean ± SD	3.4 ± 3.3	4.0 ± 3.2	4.0 ± 3.0	3.5 ± 3.1	4.2 ± 3.2	4.2 ± 3.5	4.5 ± 3.4	3.9 ± 3.5	3.1 ± 3.7	3.2 ± 3.0
	Median (IQR)	3 (1–4)	3 (2–5)	3 (2–5)	3 (2–5)	3 (2–5)	3 (2–6)	4 (2–6)	3 (2–5)	2 (1–4)	3 (1–4)
Control variables											
Age at diagnosis	Mean ± SD	61.9 ± 13.6	53.2 ± 12.6	51.8 ± 11.2	54.3 ± 13.1	53.1 ± 13.0	53.4 ± 12.2	54.6 ± 13.1	52.7 ± 13.0	52.1 ± 11.2	53.7 ± 13.3
Co-morbidities (ADGs)	0 -- 5	20,321 (49.0)	2760 (50.2)	823 (50.3)	495 (53.1)	340 (45.2)	252 (49.1)	471 (48.7)	132 (50.6)	69 (57.0)	111 (35.7)
	6 -- 9	14,942 (36.0)	1994 (36.3)	617 (37.7)	320 (34.3)	309 (41.1)	175 (34.1)	355 (36.7)	95 (36.4)	37 (30.6)	86 (27.7)
	10 +	6193 (14.9)	741 (13.5)	196 (12.0)	118 (12.6)	103 (13.7)	86 (16.8)	142 (14.7)	34 (13.0)	15 (12.4)	47 (15.1)
Income quintile	1 (lowest)	6720 (16.2)	1411 (25.7)	414 (25.3)	210 (22.5)	232 (30.9)	124 (24.2)	264 (27.3)	91 (34.9)	17 (14.0)	59 (19.0)
	2	7917 (19.1)	1173 (21.3)	387 (23.7)	167 (17.9)	206 (27.4)	81 (15.8)	214 (22.1)	39 (14.9)	15 (12.4)	64 (20.6)
	3	8006 (19.3)	1099 (20.0)	330 (20.2)	160 (17.1)	147 (19.5)	102 (19.9)	239 (24.7)	55 (21.1)	20 (16.5)	46 (14.8)
	4	9017 (21.8)	1032 (18.8)	287 (17.5)	218 (23.4)	100 (13.3)	112 (21.8)	175 (18.1)	42 (16.1)	27 (22.3)	71 (22.8)
	5	9646 (23.3)	776–780	211–215	176–180	67 (8.9)	94 (18.3)	76 (7.9)	34 (13.0)	42 (34.7)	71 (22.8)
	Missing	150 (0.4)	<=5	<=5	<=5	0 (0.0)	0 (0.0)	0 (0.0)	0 (0.0)	0 (0.0)	0 (0.0)
Outcome variables											
Stage at diagnosis	I	15,732 (37.9)	1736 (31.6)	586 (35.8)	294 (31.5)	197 (26.2)	164 (32.0)	250 (25.8)	84 (32.2)	52 (43.0)	109 (35.0)
	II	14,624 (35.3)	1983 (36.1)	602 (36.8)	338 (36.2)	288 (38.3)	197 (38.4)	321 (33.2)	95 (36.4)	35 (28.9)	107 (34.4)
	III	5271 (12.7)	837 (15.2)	196 (12.0)	176 (18.9)	131 (17.4)	73 (14.2)	161 (16.6)	38 (14.6)	18 (14.9)	44 (14.1)
	IV	1849 (4.5)	210 (3.8)	43 (2.6)	38 (4.1)	43 (5.7)	13 (2.5)	42 (4.3)	13 (5.0)	6 (5.0)	12 (3.9)
	Unknown	3980 (9.6)	729 (13.3)	209 (12.8)	87 (9.3)	93 (12.4)	66 (12.9)	194 (20.0)	31 (11.9)	10 (8.3)	39 (12.5)
Screen detected	n (%) screen-detected	11,876 (28.6)	1081 (19.7)	295 (18.0)	191 (20.5)	131 (17.4)	121 (23.6)	194 (20.0)	52 (19.9)	28 (23.1)	69 (22.2)
Diagnostic Interval (Screened patients)	Median (IQR)	28 (15–47)	33 (19–55)	38 (23–60)	31 (17–49)	33 (21–58)	30 (20–57)	33 (19–50)	33 (21–49)	22 (9–35)	28 (13–48)
	90th percentile	73	86	91	76	99	107	84	74	76	107
Diagnostic Interval (Symptomatic patients)	Median (IQR)	33 (15–66)	38 (19–76)	44 (22–82)	35 (16–73)	41 (21–78)	32 (15–73)	35 (17–64)	40 (19–77)	32 (16–68)	36 (15–77)
	90th percentile	126	130	136	127	140	120	118	112	142	133

*P*-value <.001 for all variables for comparison of immigrants versus long-term residents

In multivariate analysis, immigrant women as a group were not significantly different than long-term residents for stage I/II vs. stage III/IV of diagnosis in either province (Table 4). However, women from East Asia and the Pacific were significantly more likely to be diagnosed at stage I/II than long-term residents in both provinces (adjusted relative risk with 95% confidence interval: 1.28 [1.08–1.52] in BC, 1.05 [1.03–1.08] in Ontario). In Ontario, women were significantly less likely to be diagnosed at stage I/II if they were from Eastern Europe and Central Asia, Latin America and the Caribbean, or South Asia. Although not statistically significant, the BC point estimates for these same groups were in the same direction as the Ontario results. In BC, women from the economic immigrant class were significantly more likely to be diagnosed at stage I/II (adjusted relative risk or ARR with 95% confidence interval or CI: 1.20 [1.02–1.42]), while women from the family class trended toward a lesser likelihood of stage I/II diagnosis. Time in Canada was not significantly associated with stage of diagnosis in either province.

**Table 4:** Adjusted relative risk (ARR) of stage I/II vs. stage III/IV (reference) stage at diagnosis^a^

	BC	Ontario
Immigrants overall	**ARR [95% CI]**	**ARR [95% CI]**
Long-term residents	0.96 [0.85–1.09]	0.98 [0.97–1.00]
Region of Origin	1.0	1.0
East Asia & Pacific	**1.28 [1.08–1.52]**	**1.05 [1.03–1.08]**
Eastern Europe & Central Asia	0.86 [0.59–1.26]	**0.94 [0.91–0.98]**
Latin America & Caribbean	0.98 [0.57–1.70]	**0.93 [0.89–0.97]**
Middle East & North Africa	0.72 [0.48–1.09]	1.01 [0.97–1.06]
South Asia	0.79 [0.58–1.06]	**0.93 [0.89–0.97]**
Sub-Saharan Africa	0.80 [0.47–1.36]	0.99 [0.92–1.05]
USA/New Zealand/Australia	0.91 [0.53–1.57]	0.98 [0.89–1.09]
Western Europe	0.86 [0.60–1.24]	1.01 [0.95–1.07]
Long-term residents	1.0	1.0
Immigrant Class	**ARR [95% CI]**	**ARR [95% CI]**
Economic	**1.20 [1.02–1.42]**	1.00 [0.98–1.03]
Family	0.87 [0.73–1.04]	0.97 [0.95–1.00]
Refugee	0.98 [0.62–1.54]	0.96 [0.92–1.00]
Other	0.68 [0.28–1.65]	1.00 [0.91–1.11]
Long-term residents	1.0	1.0
Time in Canada	**ARR [95% CI]**	**ARR [95% CI]**
Less than 10 years	0.98 [0.81–1.17]	0.99 [0.96–1.02]
10 years or more	1.08 [0.92–1.26]	0.98 [0.96–1.00]
Long-term residents	1.0	1.0

^a^Separate analyses were run stratifying immigrant women by 1) region of origin, 2) immigrant class and 3) time in Canada. Control variables in all models were age at diagnosis, level of co-morbidity and neighbourhood income quintile. Significant results are bolded

Immigrant women overall were significantly less likely to have a screen-detected breast cancer in both provinces (ARR with 95% CI: 0.88 [0.79–0.96] in BC, 0.88 [0.84–0.93] in Ontario) and women from East Asia and the Pacific were significantly less likely to have a screen-detected cancer in both provinces (Table 5). In Ontario, women were significantly less likely to have a screen-detected cancer if they were from Latin America and the Caribbean (ARR with 95% CI: 0.80 [0.69–0.94]) or from South Asia (ARR with 95% CI: 0.80 [0.71–0.91]). In BC, although marginally significant, the point estimate was in the opposite direction from Ontario for Latin American/Caribbean women. In both provinces, arriving via family class and being in Canada for less than 10 years were significantly associated with a lower likelihood of screen-detected cancer, with those in Canada 10 years or more in Ontario also having fewer screen-detected cancers.

**Table 5:** Adjusted relative risk (ARR) of having a screen-detected breast cancer^a^

	BC	Ontario
Immigrants overall	**ARR [95% CI]**	**ARR [95% CI]**
Long-term residents	**0.88 [0.79–0.96]**	**0.88 [0.84–0.93]**
Region of Origin	1.0	1.0
East Asia & Pacific	**0.85 [0.75–0.97]**	**0.86 [0.78–0.95]**
Eastern Europe & Central Asia	0.86 [0.61–1.22]	0.89 [0.79–1.01]
Latin America & Caribbean	1.34 [0.92–1.96]	**0.80 [0.69–0.94]**
Middle East & North Africa	0.96 [0.67–1.38]	1.00 [0.86–1.16]
South Asia	0.96 [0.74–1.23]	**0.80 [0.71–0.91]**
Sub-Saharan Africa	0.88 [0.54–1.43]	0.94 [0.75–1.18]
USA/New Zealand/ Australia	0.67 [0.41–1.10]	1.00 [0.74–1.35]
Western Europe	0.77 [0.55–1.08]	1.09 [0.90–1.32]
Long-term residents	1.0	1.0
Immigrant Class	**ARR [95% CI]**	**ARR [95% CI]**
Economic	0.91 [0.80–1.03]	0.93 [0.86–1.00]
Family	**0.81 [0.69–0.95]**	**0.83 [0.76–0.90]**
Refugee	0.95 [0.66–1.36]	0.88 [0.76–1.01]
Other	1.09 [0.41–2.92]	0.94 [0.66–1.33]
Long-term residents	1.0	1.0
Time in Canada	**ARR [95% CI]**	**ARR [95% CI]**
Less than 10 years	**0.80 [0.68–0.95]**	**0.76 [0.69–0.85]**
10 years or more	0.91 [0.81–1.02]	**0.94 [0.88–0.99]**
Long-term residents	1.0	1.0

^a^Separate analyses were run stratifying immigrant women by 1) region of origin, 2) immigrant class and 3) time in Canada. Control variables in the models were age at diagnosis, level of co-morbidity and neighbourhood income quintile. Significant results are bolded

In quantile regression analyses, the adjusted median diagnostic interval was 2 days longer in BC and 5.5 days longer in Ontario for immigrant women overall (Table 6). The adjusted difference in the diagnostic interval was consistently longer in Ontario than in BC across groups. Women from East Asia and the Pacific and from South Asia had significantly longer diagnostic intervals at the median in both provinces, reaching 9.5 days for East Asian women in Ontario. Ontario women from Latin America and the Caribbean also waited 9 days longer than long-term residents. In BC, only women in the family class had a statistically significantly longer wait than long-term residents at 4 days, while in Ontario, all three class groups had the same result as the immigrant group as a whole.

**Table 6:** Adjusted difference in length of diagnostic interval measured in days at the 50th percentile, where the outcome was the length of the diagnostic interval. Long-term residents served as the reference group for each analysis^a^

	BC	Ontario
	Median [with 95% confidence intervals]	Median [with 95% confidence intervals]
Immigrants overall	**2 [0.2–3.8]**	**5.5 [4.4–6.6]**
Region of origin		
East Asia & Pacific	**2 [0.1–3.9]**	**9.5 [7.1–11.9]**
Eastern Europe & Central Asia	0.4 [−5.5–6.3]	**4.5 [2.0–7.0]**
Latin America & Caribbean	2.2 [−6.9–11.2]	**9.0 [5.7–12.3]**
Middle East & North Africa	-2 [−10.9–6.9]	1.8 [−2.4–6.0]
South Asia	**6.7 [2.0–11.3]**	**3.5 [1.1–5.9]**
Sub-Saharan Africa	1.0[−14.6–16.6]	5.5 [− 1.6–12.6]
USA/New Zealand/ Australia	−0.3[− 12.2–11.5]	−2.5 [−7.2–2.2]
Western Europe	− 5[− 10.8–0.8]	1.2 [−4.5–6.9]
Immigrant class		
Economic	1[− 1.3–3.3]	**5.5 [3.8–7.2]**
Family	**4 [0.6–7.4]**	**5.5 [3.9–7.1]**
Refugee	0.2[−9.2–9.6]	**5.5 [2.1–8.9]**
Other	−9.5[−48.3–29.3]	7 [−0.4–14.4]
Time in Canada		
Less than 10 years	1.4[−1.7–4.4]	**5.5 [3.6–7.4]**
10 years or more	2[−0.3–4.3]	**5.5 [4.1–6.9]**

^a^Separate analyses were run stratifying immigrant women by 1) region of origin, 2) immigrant class and 3) time in Canada. Control variables in the models were age at diagnosis, stage at diagnosis, level of co-morbidity and neighbourhood income quintile. Significant results are bolded

In post hoc analyses, for both provinces, the interaction between years since immigration and immigrant class was statistically significant (*p* < 0.0001) for screening, which justified our analytic approach.

## Discussion

In this population-based retrospective cohort study in two Canadian provinces with large foreign-born populations, we have shown that there are inequalities in breast cancer diagnosis for immigrant women. In both provinces, immigrant women overall were significantly less likely to have a screen-detected breast cancer and had a significantly longer median diagnostic interval than long-term residents after adjusting for important control variables. However, we found significant subgroup variation when we characterized immigrant status in different ways. In both provinces, women from East Asia and the Pacific, the largest immigrant group, were less likely to have a screen-detected cancer and had a longer diagnostic interval (after adjusting for stage of diagnosis), but were diagnosed at an earlier stage than long-term residents. In Ontario, both women from Latin America and the Caribbean and women from South Asia were less likely to have a screen-detected cancer, had a longer median diagnostic interval after adjusting for stage, and were diagnosed at a later stage than long-term residents. Immigrant class and time in Canada were also associated with breast cancer diagnosis, with women in the family class being at higher risk of having breast cancers that were not screen-detected, of having longer median diagnostic intervals, and of being diagnosed at a later stage. In both provinces, women in Canada for less than 10 years were less likely to have a screen-detected cancer with evidence of a dose response for time in Canada. The differences we found do not seem to be explained by lack of primary care access, as immigrant women and long-term residents made a similar number of primary care visits during the six months prior to diagnosis. However, it is noteworthy that immigrant women had a lower prevalence of a high UPC index.

Our findings that immigrant women from certain world regions were less likely to have a screen-detected breast cancer are consistent with existing literature whereby some immigrant women in Canada have been found to have lower uptake of screening mammography [6, 9–13]. Vahabi et al.’s Ontario study found South Asian women, the most recent immigrant women, and women in the family and refugee classes to be particularly at risk of under-screening [6], in line with our findings. Surprisingly, in that same study, women from Latin America and the Caribbean had higher screening rates than long-term residents [6], yet, we found them to be less likely to have a screen-detected breast cancer. The reasons for this cannot be determined from the current study but are not out of line with US literature. African-American women have both similar screening rates and a higher incidence of aggressive breast cancers compared to White American women [37–41], and most African-American women and many Caribbean and Latin American women in Ontario would be of West African ancestry. These findings taken together may reflect that women of West African ancestry are genetically more susceptible to aggressive breast cancers that are more likely to be detected by symptoms than by screening [42].

We found that women from Latin America and the Caribbean and women from South Asia tended to be diagnosed with breast cancer at a later stage, which is consistent both with our findings that they were less likely to have a screen-detected cancer and with other literature [42, 43]. However, it is surprising that East Asian women were diagnosed at an earlier stage than long-term residents, despite being *less* likely to have a screen-detected cancer. Other studies have found East Asian women to be more likely to be diagnosed at an earlier stage than other ethnocultural groups [14, 42, 43] and to have tumours that are less likely to have spread at the time of diagnosis [43, 44]. More research is needed to explore the causes for these findings, but it is important to note that variation also exists within immigrant groups; for example, Filipino women may be at increased risk of more aggressive breast cancers when compared to other East Asian women [45]. Of note, although we did not find a difference in diagnosis of stage I-II vs. III-IV for immigrant women overall, Iqbal et al. found that immigrant women overall in Ontario were significantly less likely than Canadian-born women to be diagnosed with stage I (vs. II-IV) breast cancer [43].

It is concerning that the length of the diagnostic interval was significantly longer for some immigrant women even after adjustment for stage of diagnosis and considering no differences in primary care access, and longer in Ontario than in BC. In previous literature, Black and South Asian ethnicity have been associated with a longer diagnostic interval for breast cancer in the UK, as has Hispanic and African-American ethnicity in the US, with inequitable access to appropriate, timely and high-quality care being suggested as possible causes [17, 46–49]. Although it is admittedly unlikely that a delay in the order of days would contribute to significantly worse survival, more timely diagnosis processes for patients may reduce psychological stress experienced during this period of uncertainty [50–52].

Our study is the first Canadian population-based study that we know of to use data from two provinces to explore breast cancer diagnosis for immigrant women from all major regions of the world. Using data from two provinces allowed us to highlight similar findings of immigration-related disparities in two distinct health care systems, and to highlight important differences worth exploring such as a longer adjusted difference in the length of the diagnostic interval for immigrant women in Ontario versus BC. Lipscomb et al. have noted the importance from a policy perspective of comparative analyses across health systems in cancer care [8]. We were also able to make use of an established and rich database capturing immigrants who came to Canada as long as 26 years before the study period. This rich data source allowed us to characterize the immigrant populations in different ways that will be useful for targeted screening and diagnostic service policies and program planning. However, there are several limitations. First, the IRCC database is not complete. It does not include immigrants who arrived before 1985, therefore we designated our reference group as long-term residents. It also does not include immigrants who landed in other provinces and then moved to BC or Ontario. An estimated 9.6% of immigrants residing in Ontario and 20.2% of immigrants residing in BC first landed in another province [53]. These immigrant women would have been included as long-term residents. The differences we found may have been larger if we had been able to accurately classify these interprovincial migrants. Second, the sample size was small for some immigrant subgroups in BC, and thus some of our analyses in this province were under-powered. Third, stage of diagnosis was not available for all study participants. However, there is no reason to believe that this information would be missing in a biased manner. Fourth, we did not take into account the variation that exists within immigrant groups. Woods et al. found that breast cancer screening participation rates in BC were 37.9% for women from Eastern Europe and Central Asia, but within that group, rates from individual countries ranged from 35.0 to 49.0% [29]. Collapsing birth countries into regions may also explain some of the interprovincial differences we observed. For example, according to the 2016 Canada Census, the most common Latin American and Caribbean source country for BC is Mexico, whereas for Ontario, the most common source country within that region is Jamaica. Differences for this world region in the two provinces may be partly explained by this different country mix. Fifth, it is not possible to determine the reasons for the differences we observed using available administrative data. Systemic barriers, discrimination, cultural barriers, and genetic susceptibility due to shared ancestry are all potential reasons, and it is possible that all play some role. Finally, we did not explore the impact of the differences we found in the diagnostic process on breast cancer-specific survival, which would be crucial to explore in future studies. The disparities we found in screen-detected cancers were notably larger than the disparities we found in stage of diagnosis, bringing to mind questions that have previously been raised in the literature about the role of screening mammography in breast cancer survival and in over-diagnosis [54–57].

We have shown that there are inequalities in breast cancer diagnosis for some immigrant women in the Canadian provinces of BC and Ontario. In particular, we have highlighted particular immigrant groups that appear to have unique issues that need to be explored in order to effectively reduce these gaps in care. Future research exploring these findings must particularly consider how the quality of primary care can be improved for these women, and must explore if immigrant women experience disparities in breast cancer-specific survival.

## Abbreviations

ACG: Adjusted Clinical Groups
ADG: Aggregated Diagnosis Groups
ARR: Adjusted Relative Risk
BC: British Columbia
CanIMPACT: Canadian Team to Improve Community-Based Cancer Care along the Continuum
CI: Confidence Interval
IRCC: Immigration Refugee and Citizenship Canada
UPC: Usual Provider of Care
